# A multi-parametric prognostic model based on clinical features and serological markers predicts overall survival in non-small cell lung cancer patients with chronic hepatitis B viral infection

**DOI:** 10.1186/s12935-020-01635-8

**Published:** 2020-11-19

**Authors:** Shulin Chen, Hanqing Huang, Yijun Liu, Changchun Lai, Songguo Peng, Lei Zhou, Hao Chen, Yiwei Xu, Xia He

**Affiliations:** 1 State Key Laboratory of Oncology in South China, Collaborative Innovation Center for Cancer Medicine, Guangdong Key Laboratory of Nasopharyngeal Carcinoma Diagnosis and Therapy, Sun Yat-Sen University Cancer Center, 651 Dongfeng Road East, Guangzhou, 510060 People’s Republic of China; 2 Department of Thoracic Surgery, Maoming People’s Hospital, Maoming, 525000 Guangdong People’s Republic of China; 3 Department of Clinical Laboratory, Maoming People’s Hospital, Maoming, 525000 Guangdong People’s Republic of China; 4 Department of Pathology Laboratory, Maoming People’s Hospital, Maoming, 525000 Guangdong People’s Republic of China; 5 Department of Clinical Laboratory, The Cancer Hospital of Shantou University Medical College, Precision Medicine Research Center, Shantou University Medical College, Shantou, 515041 People’s Republic of China

**Keywords:** Hepatitis B virus, Lasso regression, Model, Non-small cell lung cancer, Prognostic

## Abstract

**Background:**

To establish and validate a multi-parametric prognostic model based on clinical features and serological markers to estimate the overall survival (OS) in non-small cell lung cancer (NSCLC) patients with chronic hepatitis B viral (HBV) infection.

**Methods:**

The prognostic model was established by using Lasso regression analysis in the training cohort. The incremental predictive value of the model compared to traditional TNM staging and clinical treatment for individualized survival was evaluated by the concordance index (C-index), time-dependent ROC (tdROC) curve, and decision curve analysis (DCA). A prognostic model risk score based nomogram for OS was built by combining TNM staging and clinical treatment. Patients were divided into high-risk and low-risk subgroups according to the model risk score. The difference in survival between subgroups was analyzed using Kaplan–Meier survival analysis, and correlations between the prognostic model, TNM staging, and clinical treatment were analysed.

**Results:**

The C-index of the model for OS is 0.769 in the training cohorts and 0.676 in the validation cohorts, respectively, which is higher than that of TNM staging and clinical treatment. The tdROC curve and DCA show the model have good predictive accuracy and discriminatory power compare to the TNM staging and clinical treatment. The prognostic model risk score based nomogram show some net clinical benefit. According to the model risk score, patients are divided into low-risk and high-risk subgroups. The difference in OS rates is significant in the subgroups. Furthermore, the model show a positive correlation with TNM staging and clinical treatment.

**Conclusions:**

The prognostic model showed good performance compared to traditional TNM staging and clinical treatment for estimating the OS in NSCLC (HBV+) patients.

## Background

At present, lung cancer is the leading cause of cancer morbidity and mortality worldwide [1]. Non-small-cell lung cancer (NSCLC) accounts for 75–80% of all lung malignancies [2]. The 5-year survival of NSCLC patients is generally poor because of late diagnosis, frequent relapse, and the lack of effective systemic therapy [3].

Hepatitis B virus (HBV) is one of the most prevalent and most serious types of viral hepatitis, and the prevalence of HBV in China is high [4]. Therefore, it is reasonable to hypothesize that a HBV infection may be an important comorbidity factor in NSCLC patients in China. Previous studies have shown that HBV associated with several extra-hepatic cancers [5–7], In addition, diffuse large B-cell lymphoma [8] and multiple myeloma [9] patients with HBV infection have poor survival outcomes compared to non-infected patients. Together, these results implied that NSCLC patients with HBV infection should be distinguished from uninfected patients because they have different clinical characteristics, outcomes and prognostic factors. This may aid in the development of a distinct prognostic predictive model for NSCLC patients with HBV infection.

Currently, the TNM (tumor, lymph node, metastasis) stage is a widely used staging system for predicting the outcome of NSCLC patients [10]. However, patients within a similar TNM stage show different genetic, cellular, and clinicopathological characteristics, and exhibit a wide spectrum of clinical survival outcomes. This indicates the need for additional prognostic factors to complement the TNM staging to better predict the outcome of the NSCLC patients [11–13]. Therefore, many studies have reported some prognostic factors that might improve the predict the survival of NSCLC patients [14–16]. Together, these findings could help identify patients that would benefit from novel therapeutic strategies or, alternatively, if additional treatment methods need to be pursued.

Thus, the present retrospective study aimed to develop and validate a multi-parametric prognostic model based on clinical features and serological markers to estimate the overall survival (OS) in NSCLC HBV (+) patients and assess its incremental value to the traditional staging system and clinical treatment for the estimation of OS.

## Material and methods

### Patient selection and data collection

First diagnosed NSCLC (HBV+) patients who were treated at the Sun Yat-sen University Cancer Center (Guangzhou, China) between January 2008 and December 2010 were retrospectively enrolled in this study. This study was approved by the Hospital Ethics Committee in Sun Yat-sen University Cancer Center (Guangzhou, China). The inclusion criteria were as follows: (a) pathological evidence of NSCLC; (b) patients without pathological diagnosis or with previous or concomitant malignancies; (c) positive for hepatitis B surface antigen (HbsAg); (d) no co-infected other types of hepatitis viruses; (e) complete baseline clinical information, laboratory, and follow-up data.

The following relevant clinical and serological data were collected for each enrolled patient at the time of diagnosis and before any treatment: age, gender, family history, body mass index (BMI), tumor size, clinical treatment, Tumor Node Metastasis stage (TNM stage) [17], white blood cells (WBC), neutrophils (N), lymphocytes (L), platelet (PLT), hepatitis B surface antigen (HbsAg), hepatitis B surface antibody (HBsAb), hepatitis B envelope antigen (HBeAg), hepatitis B envelope antibody(HBeAb), hepatitis B core antibody (HBcAb), hepatitis B core antigen (HBcAb), albumin (ALB), alkaline phosphatase (ALP), apolipoprotein AI (APOA), apolipoprotein B (APOB), C-reactive protein (CRP), lactic dehydrogenase (LDH), glutamyl transpeptidase (GGT), total bilirubin (TBIL), and direct bilirubin (DBIL). The NLR represented the ratio of neutrophils to lymphocytes ratio [18]; the PLR represented the ratio of platelets to lymphocytes [18]; the SLR was the ratio of aspartate aminotransferase (AST) to alanine transaminase (ALT) [19]; ABR was the ratio of APOA to APOB [20]; CAR was the ratio of CRP to ALB ratio [21]; prognostic index (PI): score 0 for CRP 10 mg/L or less and a WBC count of 11 × 10^9^/L or less, patients with only one of these abnormalities were allocated a score of 1, and patients with an elevation of both levels were elevated were allocated a score of 2 [22]. The prognostic nutritional index (PNI) was calculated according to the following formula: Alb (g/L) + 5 × lymphocyte count × 10^9^/L: score 0 for PNI > 45; score 1 in patients with PNI ≤ 45 [23]. The Glasgow prognostic score (GPS) was classified as follows: patients with serum CRP > 10 mg/L and albumin < 35 g/L were classified as GPS 2; patients with CRP > 10 mg/L or albumin < 35 g/L were classified as GPS 1; patients with serum CRP ≤ 10 mg/mL and albumin > 35 g/L were classified as GPS 0 [24].

### Patients follow up

Follow-up of patients' survival data was obtained by means of retrieving medical records, email, and direct communication by phone. All patients were followed up until death or January 2016. The endpoint of this study was overall survival (OS), which was defined as the time interval from diagnosis to the date of the patient’s death or censored at the date of the last follow-up.

### Statistical analyses

Statistical analyses were performed using IBM SPSS Statistical software version 19.0 (IBMCorp., Chicago, IL, USA) and R version 3.6.0 (http://www.R-project.org). Categorical variables were classified based on clinical findings, and continuous variables were transformed into categorical variables based on the cut-off values of by the R package "survival" [25] and "survminer". Differences in distribution between patients in the training cohort and validation cohort were analyzed by Chi-square test. The Lasso regression analysis was utilized to select the most useful prognostic variables in the training cohort. According to the regulation weight λ, LASSO shrinks all regression coefficients towards zero and sets the coefficients of many irrelevant features to zero. The optimal values of the penalty parameter λ were determined by tenfold cross validation with the 1 standard error of the minimum criteria (the 1-SE criteria), where the final value of λ yielded a minimum cross validation error. Retained features with nonzero coefficients were used for regression model fitting [26, 27]. Next, a prognostic computing-based model was established for each patient through a linear combination of selected variables weighted by their respective coefficients. The R package "glmnet" was used for Lasso regression analysis. The incremental predictive value of the prognostic model to the traditional TNM staging and clinical treatment for individualized survival was evaluated by the Harrell's concordance index (C-index), time-dependent ROC (tdROC), and decision curve analysis [28]. The area under the curve (AUC) was calculated using the "survivalROC" package [29], and the C-index was computed and compared by using the "survcomp" package [30]. A nomogram (by the package of rms in R) was developed using the prognostic model risk score, TNM staging, and clinical treatment. Performance was assessed by the calibration curve in internal validation with bootstrapping (1000 bootstrap resamples) [31]. For subsequent comparison, patients were divided into high- and low-risk groups basing on the optimal cut-off value of the prognostic model risk score, and Kaplan–Meier survival analyses and log-rank tests were used to assess differences in OS between patients in the predicted high- and low-risk groups. The correlation between the prognostic model and TNM staging or clinical treatment was evaluated by the Pearson's correlation coefficient [32]. Results with two-sided p values of < 0.05 were considered statistically significant.

## Results

### Patient characteristics

In this study, a total of 201 eligible patients are analyzed: 145 cases in the training cohort and 56 cases in the validation cohort. The median follow-up is 29.0 months (interquartile range (IQR):12.0–64.0) in the training cohort and 32.5 months (IQR: 11.0–60.75) in the validation cohort. The 1-, 3-, and 5-year OS rates in the training cohort are 75.2%, 46.9%, and 31.7%, respectively, and the 1-, 3-, and 5-year OS rates in the validation are 73.2%, 42.9%, and 26.8%, respectively.

The optimal cut-off value for each continuous variable is as follows: age (40 years), BMI (22.3 kg/m^2^), tumor size (4.0 cm), WBC (10.8 10^9^/L), N (8.1 10^9^/L), L (1.74 10^9^/L), PLT (163.0 10^9^/L), NLR (2.7), PLR (108.6), ALB (42.5 g/L), ALT (13.7 U/L), AST (32.2 U/L), SLR (1.5), ALP (69.6 U/L), APOA (1.2 g/L), APOB (1.0 g/L), ABR (0.8), CRP (6.2 mg/L), CAR (0.16), LDH (230.3 U/L), GGT (44.2 U/L), TBIL (15.4 μmol/L), DBIL (3.0 μmol/L), and PNI (48.1). The details regarding patients' clinical characteristics and serological markers are listed in Table 1. No clinical and serological parameters, except for ALB, PLR, HBeAg, HBeAb, and HBcAb have a significantly different distribution in the training cohort and validation cohort.

**Table 1:** Demographics and clinical characteristics of patients in the training and validation cohort

Characteristic	Training cohort	Validation cohort	*P* value
	n = (145)	n = (56)	
	No. (%)	No. (%)	
Gender			0.811
Male	109 (75.2%)	43 (76.8%)	
Female	36 (24.8%)	13 (23.2%)	
Age (years)			0.431
≤ 40	15 (10.3%)	8 (14.3%)	
> 40	130 (89.7%)	48(85.7%)	
Family history			0.898
Yes	35 (24.1%)	14 (25.0%)	
No	110 (75.9%)	42 (75.0%)	
Smoking behavior			0.819
Yes	88 (60.7%)	33 (58.9%)	
No	57 (39.3%)	23 (41.1%)	
BMI (kg/m^2^)			0.630
≤ 22.3	80 (55.2%)	33 (58.9%)	
> 22.3	65 (44.8%)	23 (41.1%)	
TNM stage^a^			0.846
I	34 (23.4%)	11 (19.6%)	
II	14 (9.7%)	5 (8.9%)	
III	50 (34.5%)	23 (41.1%)	
IV	47 (32.4%)	17 (30.4%)	
Size (cm)^b^			0.911
≤ 4.0	79 (54.5%)	31 (55.4%)	
> 4.0	66 (45.5%)	25 (44.6%)	
Treatment			0.648
Sur	31 (21.4%)	13 (23.2%)	
Sur and Rad/Che	47 (32.4%)	18 (32.1%)	
Rad/Che	54 (37.2%)	17 (30.4%)	
Other	13 (9.0%)	8 (14.3%)	
WBC (10 ^9^/L)			0.560
≤ 10.8	125 (86.2%)	50 (89.3%)	
> 10.8	20 (13.8%)	6 (10.7%)	
Neutrophils (10^9^/L)			
≤ 8.1	128 (88.3%)	51 (91.1%)	0.569
> 8.1	17 (11.7%)	5 (8.9%)	
Lymphocyte (10^9^/L)			
≤ 1.74	40 (27.6%)	17 (30.4%)	0.696
> 1.74	105 (72.4%)	39 (69.6%)	
Platelet (10^9^/L)			0.245
≤ 163.0	22 (15.2%)	5 (8.9%)	
> 163.0	123 (84.8%)	51 (91.1%)	
NLR			0.521
≤ 2.7	90 (62.1%)	32 (57.1%)	
> 2.7	55 (37.9%)	24 (42.9%)	
PLR			0.043
≤ 108.6	64 (44.1%)	16 (28.6%)	
> 108.6	81 (55.9%)	40 (71.4%)	
HBsAb			0.107
Negative	145 (100.0%)	55 (98.2%)	
Positive	0 (0.0%)	1 (1.8%)	
HBeAg			0.026
Negative	142 (97.9%)	51 (91.1%)	
Positive	3 (2.1%)	5 (8.9%)	
HBeAb			0.016
Negative	8 (5.5%)	9 (16.1%)	
Positive	137 (94.5%)	47 (83.9%)	
HBcAb			0.022
Negative	0 (0.0%)	2 (3.6%)	
Positive	145 (100.0%)	54 (96.4%)	
ALB (g/L)			0.035
≤ 42.5	91 (62.8%)	26 (46.4%)	
> 42.5	54 (37.2%)	30 (53.6%)	
ALT (U/L)			0.260
≤ 13.7	26 (17.9%)	14 (25.0%)	
> 13.7	119 (82.1%)	42 (75.0%)	
AST (U/L)			0.370
≤ 32.2	124 (85.5%)	45 (80.4%)	
> 32.2	21 (14.5%)	11 (19.6%)	
SLR			0.987
≤ 1.5	127 (87.6%)	49 (87.5%)	
> 1.5	18 (12.4%)	7 (12.5%)	
ALP (U/L)			0.931
≤ 69.6	56 (38.6%)	22 (39.3%)	
> 69.6	89 (61.4%)	34 (60.7%)	
APOA (g/L)			0.425
≤ 1.2	79 (54.5%)	27 (48.2%)	
> 1.2	66 (45.5%)	29 (51.8%)	
APOB (g/L)			0.315
≤ 1.0	101 (69.7%)	43 (76.9%)	
> 1.0	44 (30.3%)	13 (23.2%)	
ABR			0.057
≤ 0.8	14 (9.7%)	1 (1.8%)	
> 0.8	131 (90.3%)	55 (98.2%)	
CRP (mg/L)			0.443
≤ 6.2	82 (56.6%)	35 (62.5%)	
> 6.2	63 (43.6%)	21 (37.5%)	
CAR			0.278
≤ 0.16	81 (17.9%)	36 (64.3%)	
> 0.16	64 (44.1%)	20 (35.7%)	
LDH (U/L)			0.755
≤ 230.3	119 (82.1%)	47 (83.9%)	
> 230.3	26 (17.9%)	9 (16.1%)	
GGT (U/L)			0.731
≤ 44.2	116 (80.0%)	46 (82.1%)	
> 44.2	29 (20.0%)	10 (17.9%)	
TBIL (umol/L)			0.652
≤ 15.4	115 (79.3%)	46 (82.1%)	
> 15.4	30 (20.7%)	10 (17.9%)	
DBIL (umol/L)			0.813
≤ 3.0	57 (39.3%)	21 (37.5%)	
> 3.0	88 (60.7%)	35 (62.5%)	
PNI			0.084
≤ 48.1	34 (23.4%)	7 (12.5%)	
> 48.1	111 (76.6%)	49 (87.5%)	
PI			0.521
0	92 (63.4%)	40 (71.4%)	
1	39 (26.9%)	11 (19.7%)	
2	14 (9.7%)	5 (8.9%)	
GPS			0.456
0	93 (64.1%)	41 (73.2%)	
1	46 (31.7%)	13 (23.2%)	
2	6 (4.1%)	2 (3.6%)	

*BMI* body mass index, *TNM* tumor node metastasis stage, *Sur* surgery; Rad: radiotherapy, *Che* chemotherapy, *WBC* white blood cell, *NLR* neutrophil/lymphocyte ratio, *PLR* platelet/lymphocyte ratio, *ALB* albumin, *ALT* alanine transaminase, *AST* aspartate aminotransferase, *SLR* AST/ALT ratio, *ALP* alkaline phosphatase, *APOA* apolipoprotein AI, *APOB* apolipoprotein B, *ABR* APOA/APOB ratio, *CRP* C-reactive protein, *CAR* C-reactive protein/albumin ratio, *LDH* lactic dehydrogenase, *GGT* glutamyl transpeptidase, *TBIL* total bilirubin, *DBIL* direct bilirubin, *PNI* prognostic nutritional index, *PI* prognostic index, *GPS* Glasgow prognostic score

^a^TNM stage was classified according to the AJCC 7th TNM staging system

^b^The tumor maximum diameter

### Construction of the multi-parametric prognostic model based on clinical and serological markers

**Fig. 1:**
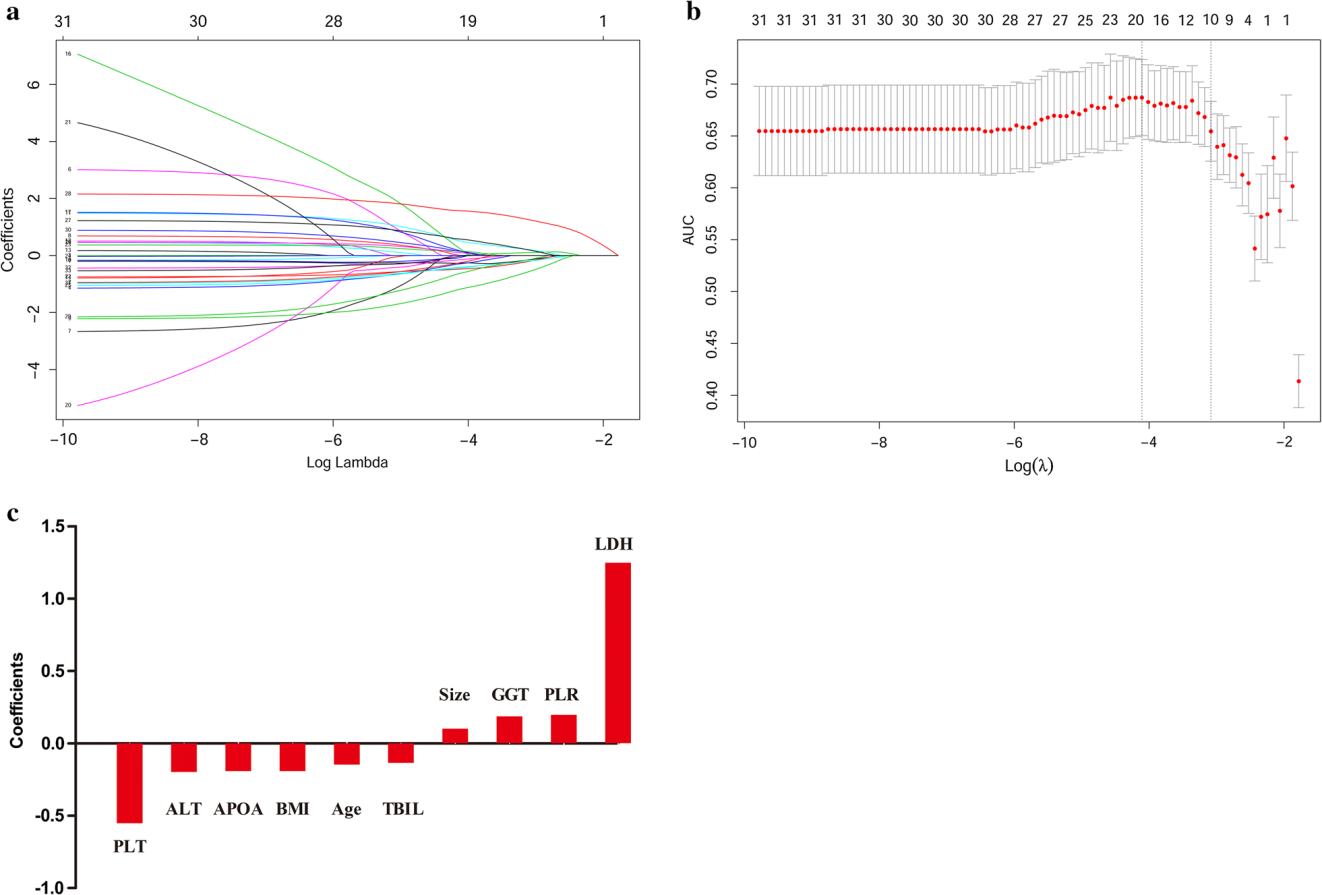
Potential predictors selection using Lasso regression analysis. **a** The changing trajectory of each predictor. The horizontal axis represents the log value of the each predictor λ, and the vertical axis represents the coefficient of the independent predictor; **b** Tuning the penalty parameter in Lasso regression analysis using tenfold cross validation and 1 standard error of the minimum criteria; **c** Histogram shows the role of each predictor that contribute to the developed prognostic model. The predictors that contribute to the prognostic model are plotted on the x-axis, with their coefficients in the Lasso regression analysis plotted on the y-axis

To select prognostic clinical and serological markers, the Lasso regression analysis is performed based on the OS in the training cohort. Figure 1a shows the change in trajectory for each factor analyzed. Moreover, tenfold cross-validation is used for model establishment, and the confidence interval under each λ is presented in Fig. 1b. The optimal value of λ is 0.046 in the Lasso regression analysis. Thus, this value is selected as the final model, and including 10 predictors from the 34 markers that are significant weighted prognostic factors: age, BMI, tumor size, PLT, PLR, ALT, GGT, LDH, TBIL, and APOA. The coefficients of the 10 predictors are presented in Fig. 1c. Subsequently, a multi-parametric prognostic model based on clinical and serological markers is constructed using the coefficients derived from the Lasso regression analysis. Next, a prognostic model risk score is calculated based on the personalized levels of the 10 predictors, by using the following formula: the prognostic model risk score = 0.679 − (0.148 × age) − (0.193 × BMI + (0.101 × tumor size) − (0.554 × PLT) + (0.197 × PLR) − (0.199 × ALT) + (0.186 × GGT) + (1.248 × LDH) − (0.137 × TBIL) − (0.194 × APOA). In this formula, each variable level is valued as 0 or 1; a value of 0 is assigned when the marker is less than or equal to the corresponding cut-off value, otherwise a value of 1 is assigned.

### Assessment of performance of prognostic model and verification

The C-index is used to estimate the discrimination performance between the prognostic model and TNM staging or clinical treatment. The results are presented in Table 2. In the training cohort, the C-index for the prognostic model is 0.769 (95% confidence interval (CI) 0.721–0.817), which is higher than that of TNM staging (0.710, 95% CI 0.661–0.758, P = 0.079), and clinical treatment (0.694, 95% CI 0.643–0.746, P = 0.017). Moreover, then compare to either the TNM staging or the clinical treatment, the prognostic model shows a better discrimination capability in the validation cohort with higher C-indexes.

**Table 2:** The C-index of our model, TNM staging and Treatment for prediction of OS in the training cohort and validation cohort

Factors	C-index (95% CI)	*P*
For training cohort		
Our model	0.769 (0.721–0.817)	
TNM staging	0.710 (0.661–0.758)	
Treatment	0.694 (0.643–0.746)	
Our model + TNM staging	0.784 (0.739–0.830)	
Our model vs. TNM staging		0.079
Our model vs. treatment		0.017
Our model vs. our model + TNM staging		0.218
For validation cohort		
Our model	0.676 (0.556–0.796)	
TNM staging	0.654 (0.552–0.755)	
Treatment	0.647 (0.517–0.777)	
Our model + TNM staging	0.712 (0.614–0.809)	
Our model vs. TNM staging		0.761
Our model vs. treatment		0.754
Our model vs. our model + TNM staging		0.205

*C-index* concordance index, *CI* confidence interval

P values are calculated based on normal approximation using function rcorrp.cens in Hmisc package

**Fig. 2:**
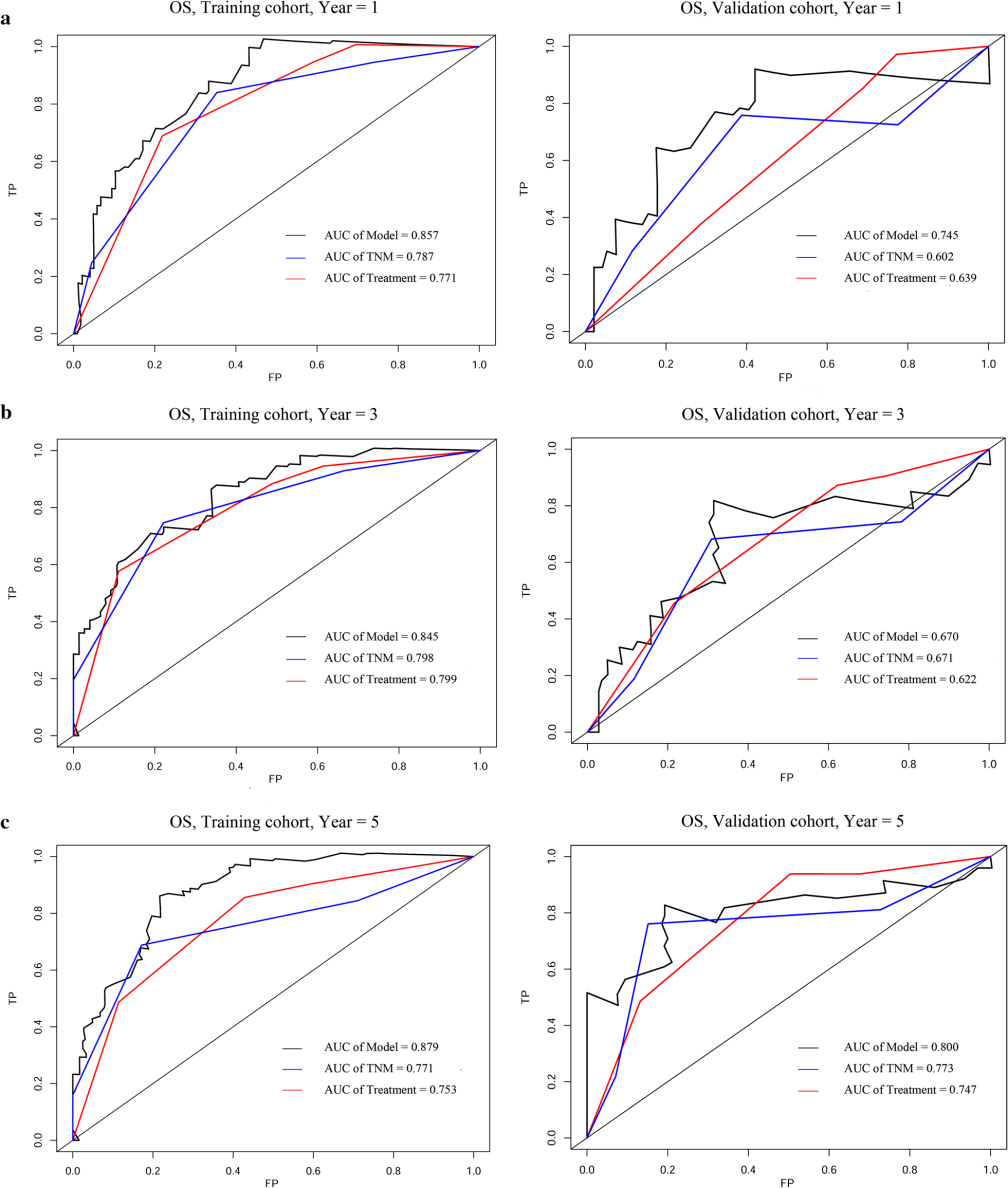
Comparison of predictive accuracy between prognostic model, TNM staging, and clinical treatment using time dependent ROC curves at 1-, 3-, 5-year (**a**–**c**) in training cohort (left) and validation cohort (right)

The prognostic accuracy of the prognostic model and TNM staging or clinical treatment in these cohorts is also assessed using tdROC analysis (Fig. 2). In the training cohort, tdROC analysis shows that the area under the ROC curves (AUCs) of the prognostic model are 0.857 for 1-year survival, 0.845 for 3-year survival, and 0.879 for 5-year survival, respectively. The AUCs of TNM staging are 0.787 for 1-year survival, 0.798 for 3-year survival, and 0.771 for 5-year survival, respectively. The AUCs of clinical treatment are 0.771 for 1-year survival, 0.799 for 3-year survival, and 0.753 for 5-year survival, respectively. Taken together, these results indicate the prognostic model have a better ability to predict survival outcomes when compare to TNM staging and clinical treatment. Similar results are observed in the validation cohort.

**Fig. 3:**
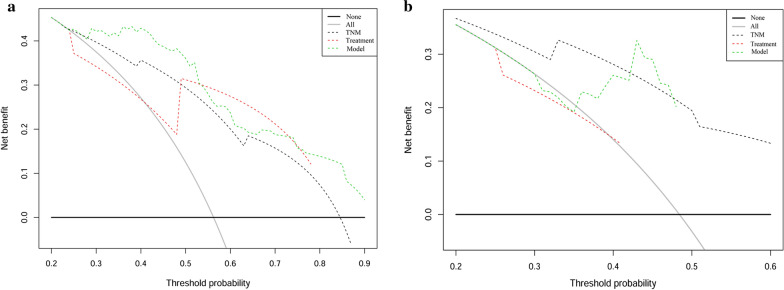
Decision curve analysis for each model in training cohort (**a**) and validation cohort (**b**). The thick grey line is the net benefit for a strategy of treating all men; the thick black line is the net benefit of treating no men. The y-axis indicate the net benefit, which is calculated by summing the benefits (true positive results) and subtracting the harms (false positive results), weighting the latter by a factor related to the relative harm of an undetected cancer compared with the harm of unnecessary treatment

In addition, decision curve analysis (Fig. 3) shows that the prognostic model have a higher overall net benefit compare to traditional TNM staging and clinical treatment across the majority of the range of reasonable threshold probabilities in the training cohort and validation cohort.

### Construction of the prognostic model risk score based nomogram

**Fig. 4:**
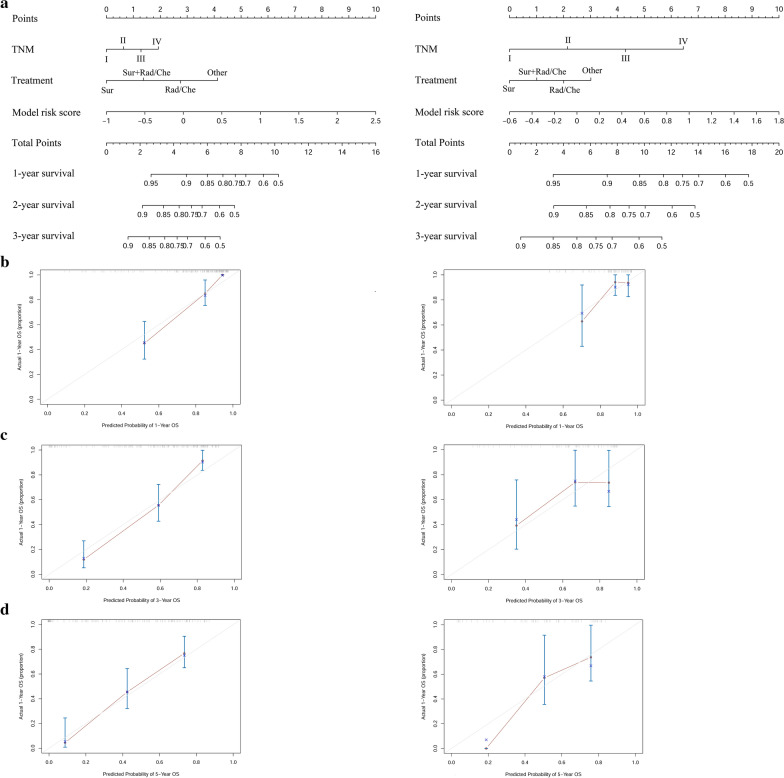
The nomograms (**a**) are used to estimate OS for NSCLC (HBV+) patients, along with the calibration plot (**b**–**d**) for the nomograms at 1-, 3-, 5- year in training cohort (left) and validation cohort (right)

In this study, we built a nomogram that consist of the prognostic model risk score, TNM staging, and clinical treatment to predict 1-, 3-, and 5-year OS in the training cohort and validation cohort (Fig. 4a). Within the variables, each subtype is assigned a point. For example, locate the patient's model risk score, draw a line straight upward to the "Points" axis to determine how many points associated with that model risk score. The process is repeated for each variable, the points achieved for each covariate are summarized, and the sum on the "Total Point" axis is located. Finally, a line is drawn straight down to identify the patient’s probability of OS at 1-, 3-, and 5-year. The calibration plots for the probability of survival at 1-, 3-, and 5-year show a good match between the prediction by the nomogram and the actual observation (Fig. 4b–d).

### Performance of the prognostic model risk score in stratifying patient risk

**Fig. 5:**
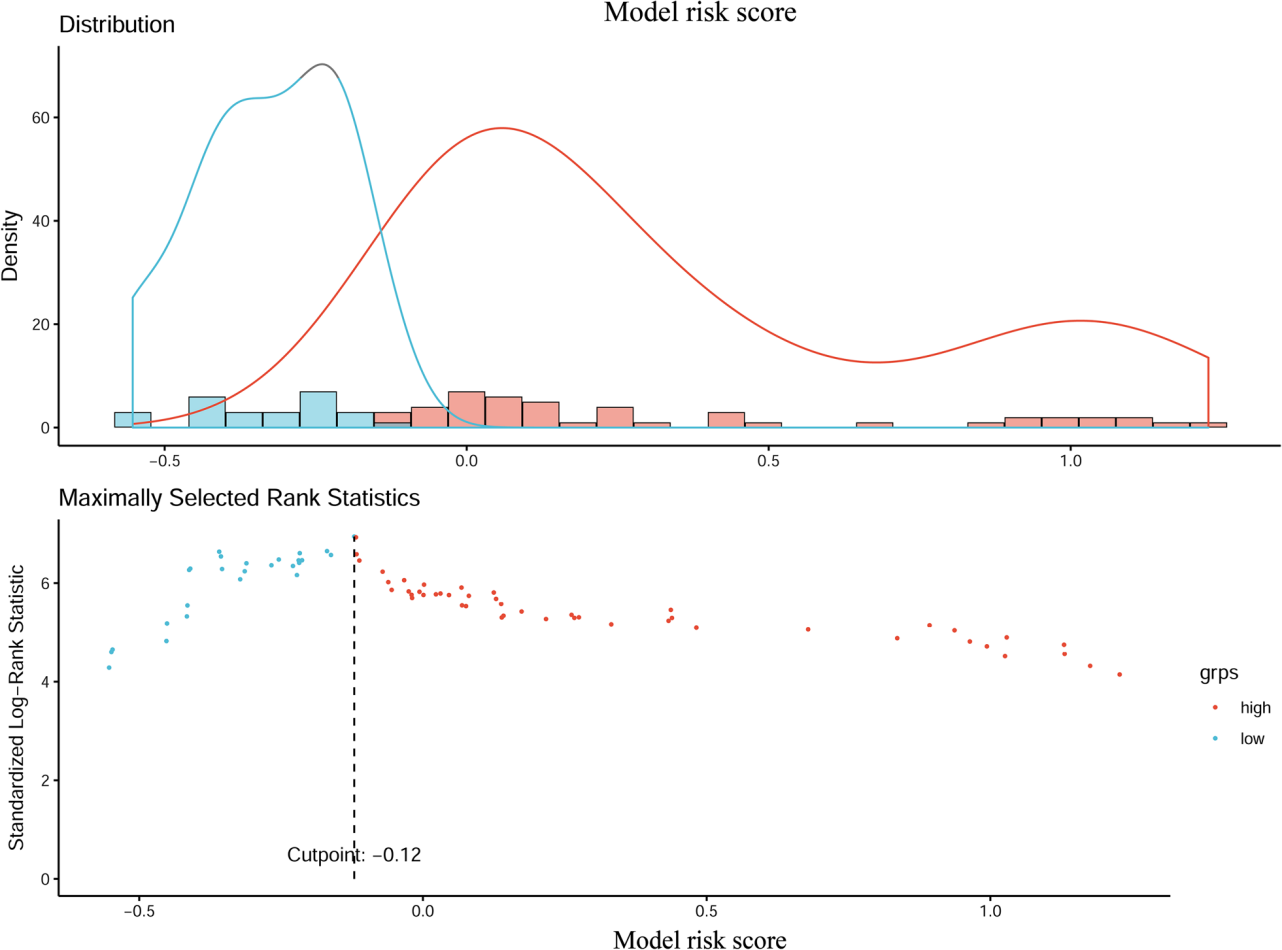
The optimal cut-off value of prognostic model risk score using R package "survival"

**Fig. 6:**
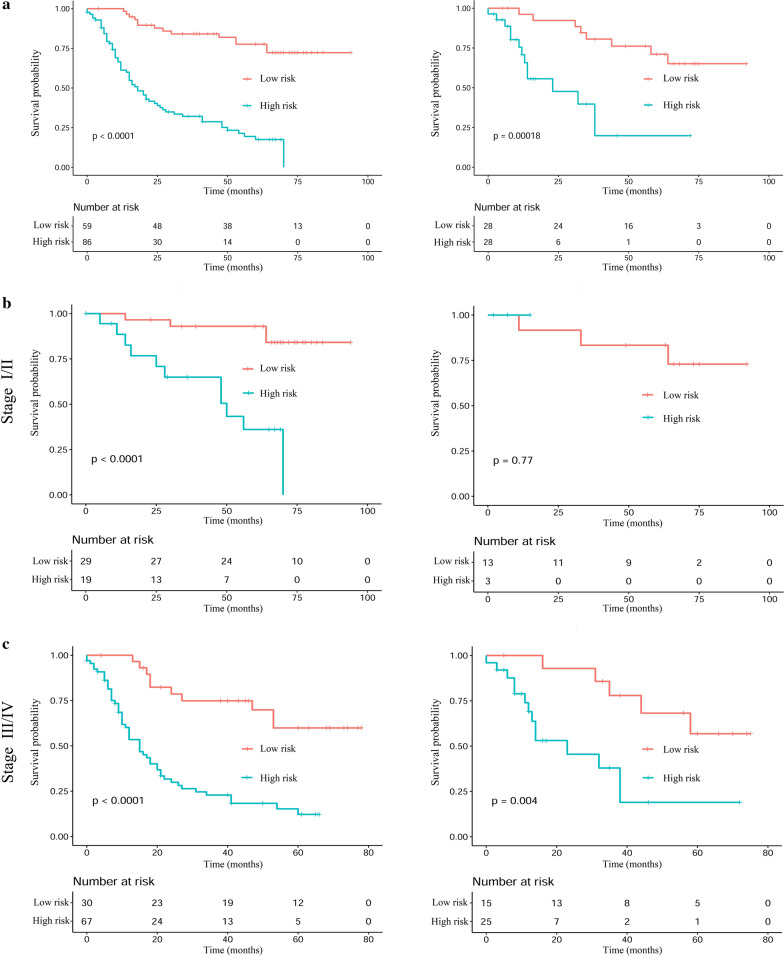
Kaplan–Meier analyses of OS according to the prognostic model risk score classifier in subgroups of NSCLC (HBV+) patients in the training cohort (left) and the validation cohort (right): (**a**) total patients; (**b**) stage I/II; (**c**) stage III/IV

The optimum cut-off value of the model risk is − 0.12 (Fig. 5). Next, patients are divided into 2 subgroups (Table 3): a low-risk group (risk score ≤ − 0.12), and a high-risk group (risk score > − 0.12). In the training cohort, for the high-risk group, the median OS of all the patients is 15 months (interquartile range (IQR): 7.0–40.0 months), the 1-, 3- and 5-year probabilities of survival are 59.3%, 26.7%, and 11.6%, respectively. For the low-risk group, the median OS is 63 months (IQR: 38.0–74.0 months), and the 1-, 3- and 5-year probabilities of survival are 98.1%, 76.3%, and 61.0%, respectively. The-low risk group have better survival probabilities compare to the high-risk group at a 1-, 3-, and 5-year survival rate. Subsequently, Kaplan–Meier survival analysis is performed according to the stratified subgroup (Fig. 6a). Kaplan–Meier curves show that significant differences are observed in survival distributions in the stratified subgroup in the training cohort. Similar results are observed in the validation cohort.

**Table 3:** OS and OS rate in high-risk and low-risk groups according to our model risk score in the training and validation cohort

Parameter	Training cohort			Validation cohort		
	High-risk group	Low-risk group	Total	High-risk group	Low-risk group	Total
No. of patients	86	59	145	28	28	56
OS						
Median	15.0	63.0		12.5	59.0	
IQR	7.0–40.0	38.0–74.0		7.25–21.50	33.50–72.25	
No. of OS						
At 1 year	51 (59.3%)	58 (98.1%)	109 (69.7%)	16 (57.1%)	25 (89.3%)	41 (73.2%)
At 3 year	23 (26.7%)	45 (76.3%)	68 (46.9%)	4 (14.3%)	20 (71.4%)	24 (42.9%)
At 5 year	10 (11.6%)	36 (61.0%)	46 (31.7%)	1 (3.6%)	14 (50.0%)	15 (26.8%)

*OS* overall survival, *IQR* interquartile range

Furthermore, stratified analyses of NSCLC HBV (+) patients with a respective stage I/II, and III/IV are performed (Fig. 6b, c). In the training cohort, the stratification by the prognostic model risk score result in significant differences in Kaplan–Meier OS curves for patients in each stage group. Furthermore, for the validation cohort, this stratification also result in significant differences in OS, except for patients in stage I/II.

### The correlation between the prognostic model and TNM staging or clinical treatment

**Fig. 7:**
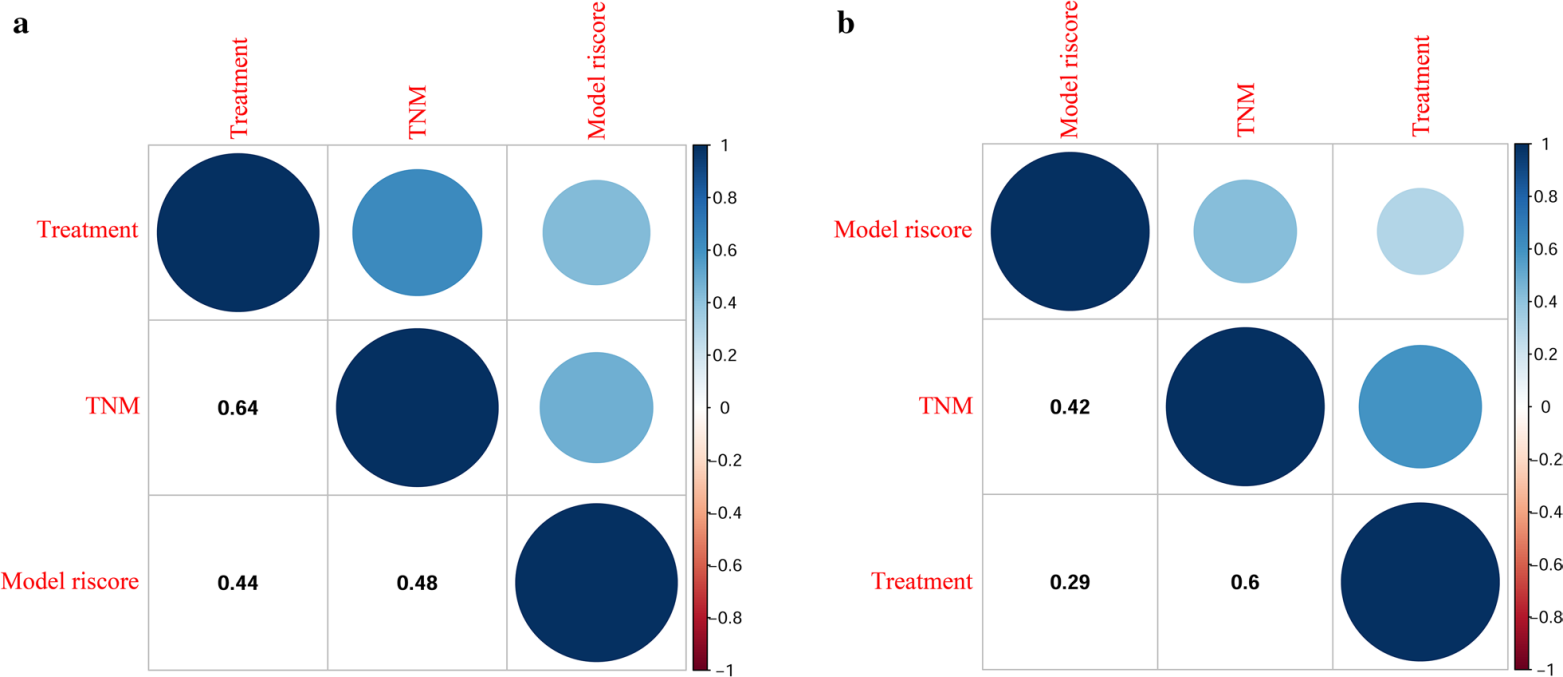
The correlations between the prognostic model and TNM staging or clinical treatment in training cohort (**a**) and validation cohort (**b**)

Figure 7 shows the correlations between the prognostic model and TNM staging or clinical treatment in the training cohort (A) and the validation cohort (B). In this plot, the blue represents positive correlations, and the red represents negative correlations. The color intensity and the size of the circle are proportional to the correlation coefficients. In addition, the numbers in the graph show the Pearson's correlation coefficient (PCC) between different variables. The results reveal that prognostic model is positively correlated with TNM staging (PCC: training cohort: 0.48; validation cohort: 0.42) and clinical treatment (PCC: training cohort: 0.44; validation cohort: 0.29).

## Discussion

In the present study, we first analyzed individual clinical features and serological markers based on the survival analysis approach. Then, a multi-parametric prognostic model was generated by using the Lasso regression model for predicting the OS in NSCLC HBV (+) patients. Our prognostic model showed better predictive accuracy and discriminative ability compared to traditional TNM staging and clinical treatment. The prognostic model signature successfully stratified those patients into high-risk and low-risk subgroups with significant differences in OS.

According to the results of Lasso regression analysis, the present prognostic model consisted of 10 prognostic factors: age, BMI, tumor size, PLT, PLR, ALT, GGT, LDH, TBIL, and APOA. Of the 10 prognosis-specific factors, all had been reported to be associated with OS in lung cancer patients [33–43]. These findings suggested that our results had credible prognostic value. We next compared the predictive accuracy of the prognostic model with the traditional TNM staging and clinical treatment. The data showed that the C-index of the prognostic model was higher compared to that of TNM staging and clinical treatment in the training cohort. TdROC curve analysis showed that our prognostic model exhibited good accuracy in clinical outcome prediction either for 1-year survival (AUC = 0.857), 3-year survival (AUC = 0.845), and 5-year survival (AUC = 0.879) of NSCLC HBV(+) patients in the training cohort when compared with traditional TNM staging and clinical treatment. Furthermore, the decision curve analysis showed that the prognostic model had good performance in prognosis prediction compared to TNM staging and clinical treatment in the training cohort. In the validation cohort, results were observed that were similar to the findings mentioned above.

To complement the shortcomings of current TNM staging in the prognostic assessment of NSCLC HBV (+) patients, the prognostic model risk score of patients was calculated, and prediction and verification were carried out. The results showed that the prognostic model risk score successfully classified patients into high-risk and low-risk subgroups within stages I/II and III/IV, and that high-risk patients had poor survival outcomes. Therefore, even between patients in the same stage, high-risk patients needed more intensified treatment. These results implied that the prognostic model could reinforce the prognostic ability of TNM staging, and the improved prediction of individual outcomes would be useful for counselling patients, personalizing treatment, and scheduling patients' follow-up. Of note, significant positive correlations were observed among the prognostic model, TNM staging, and clinical treatment, thereby suggesting that the prognostic model could be useful in predicting the outcomes of NSCLC HBV (+), and might be useful in treatment decisions.

Compared to previous studies [44, 45], this study had the following advantages: (1) To increase prognostic accuracy, many potential prognostic factors have been assessed. The potential prognostic factors included in this study were more than presented in previously studies. (2) We developed a prognostic model using the new algorithm Lasso regression analysis, as a statistical method for screening variables to establish a prognostic model, which enabled to adjust for model's over fitting and avoid extreme predictions. Thus, the predictive accuracy could be significantly improved, and this approach was applied in many study [27, 46, 47]. (3) The prognostic model was different from that presented in previous studies because the prognostic model did not include TNM staging. Therefore, whether it can be used for patients with TNM staging is unclear. Moreover, the C-index of the prognostic model was approximately equivalent or even higher than the previously reported model. (4) For further research, continuous variables need to be transformed into categorical variables based on the cut-off values. There were some limitations in choosing the cut-off values for continuous variables, because the cut-off values were determined by analyzed data, and different data have different cut-off values. To overcome this limitation, in this study, the continuous variables did not need to be transformed into categorical variables. Thus, this was convenient for other center applications.

However, some limitations in our study should be considered. First, this was a retrospective study, and therefore, the retrospective nature of this study cannot exclude all potential bias. Second, our endpoint was OS, and further research on the disease-free survival (DFS) should also be conducted. Third, other predictive biomarkers, such as radiomics features [48], carcinoembryonic antigen (CEA) [49], cytokeratin 19 fragment (CYFRA21-1) [49], epidermal growth factor receptor (EGFR) [50], circulating tumor cells [51], and circulating cell-free DNA [52] were not analyzed in the current study. Finally, analysis was from data obtained from a single cancer center, and the sample size was small. In the future, a large-scale, multicenter validation of the results will be required. Despite the above-mentioned shortcomings, the prognostic model was effective and may be useful in predicting the outcomes of NSCLC HBV (+ ) patients.

## Conclusions

In conclusion, this study provided a multi-parametric prognostic model derived from clinical features and serological markers that showed favorable performance when compared to traditional TNM staging and clinical treatment for individualized OS estimation. The nomogram based on the prognostic model, TNM staging, and clinical treatment can reinforce the prognostic ability of TNM staging. Therefore, this simple, precise and understandable prognostic model may serve as a potential tool for clinicians in counselling patients, personalizing treatment, and scheduling the follow-up for NSCLC HBV (+) patients.

## Data Availability

The datasets analyzed during the current study are not publicly available due to patient privacy concerns, but are available from the corresponding author on reasonable request.

## Abbreviations

AUC: The areas under ROC curve
BMI: Body mass index
TNM: Tumor node metastasis stage
Sur: Surgery
Rad: Radiotherapy
Che: Chemotherapy
WBC: White blood cell
NLR: Neutrophil/lymphocyte ratio
PLR: Platelet/lymphocyte ratio
ALB: Albumin
ALT: Alanine transaminase
AST: Aspartate aminotransferase
SLR: AST/ALT ratio
ALP: Alkaline phosphatase
APOA: Apolipoprotein AI
APOB: Apolipoprotein B
ABR: APOA/APOB ratio
CRP: C-reactive protein
CAR: C-reactive protein/albumin ratio
LDH: Lactic dehydrogenase
GGT: Glutamyl transpeptidase
TBIL: Total bilirubin
DBIL: Direct bilirubin
PNI: Prognostic nutritional index
PI: Prognostic index
PCC: Pearson's correlation coefficient
GPS: Glasgow prognostic score
IQR: Interquartile range
